# Recent advances and limitations of mTOR inhibitors in the treatment of cancer

**DOI:** 10.1186/s12935-022-02706-8

**Published:** 2022-09-15

**Authors:** Eunus S. Ali, Kangkana Mitra, Shamima Akter, Sarker Ramproshad, Banani Mondal, Ishaq N. Khan, Muhammad Torequl Islam, Javad Sharifi-Rad, Daniela Calina, William C. Cho

**Affiliations:** 1 College of Medicine and Public Health, Flinders University, Bedford Park, 5042 Australia; 2 Gaco Pharmaceuticals, Dhaka, 1000 Bangladesh; 3 Faculty of Medicine and Pharmacy, Université Grenoble Alpes, Grenoble, France; 4 Department of Bioinformatics and Computational Biology, George Mason University, Fairfax, VA 22030 USA; 5 Department of Pharmacy, Ranada Prasad Shaha University, Narayanganj, 1400 Bangladesh; 6 Institute of Basic Medical Sciences, Khyber Medical University, Peshawar, 25100 Pakistan; 7 Department of Pharmacy, Life Science Faculty, Bangabandhu Sheikh Mujibur Rahman Science and Technology University, Gopalganj, 8100 Bangladesh; 8 Facultad de Medicina, Universidad del Azuay, Cuenca, Ecuador; 9 Department of Clinical Pharmacy, University of Medicine and Pharmacy of Craiova, 200349 Craiova, Romania; 10 Department of Clinical Oncology, Queen Elizabeth Hospital, Kowloon, Hong Kong China; 11 Present Address: Department of Biochemistry and Molecular Genetics, and Simpson Querrey Institute for Epigenetics, Northwestern University Feinberg School of Medicine, 303 E Superior St, Chicago, IL 60611 USA

**Keywords:** Cancer, Rapamycin, mTOR pathway, mTORC1, mTORC2, mTOR inhibitors, Targeted therapy

## Abstract

The PI3K-Akt-mechanistic (formerly mammalian) target of the rapamycin (mTOR) signaling pathway is important in a variety of biological activities, including cellular proliferation, survival, metabolism, autophagy, and immunity. Abnormal PI3K-Akt-mTOR signalling activation can promote transformation by creating a cellular environment conducive to it. Deregulation of such a system in terms of genetic mutations and amplification has been related to several human cancers. Consequently, mTOR has been recognized as a key target for the treatment of cancer, especially for treating cancers with elevated mTOR signaling due to genetic or metabolic disorders. In vitro and in vivo, rapamycin which is an immunosuppressant agent actively suppresses the activity of mTOR and reduces cancer cell growth. As a result, various sirolimus-derived compounds have now been established as therapies for cancer, and now these medications are being investigated in clinical studies. In this updated review, we discuss the usage of sirolimus-derived compounds and other drugs in several preclinical or clinical studies as well as explain some of the challenges involved in targeting mTOR for treating various human cancers.

## Introduction

Cancer refers to abnormal cell growth, which often proliferates uncontrollably and is likely to metastasize and invade neighbouring cells or tissues [1, 91, 93]. A variety of factors are associated with cancer development, including DNA mutation, accumulation of cellular stress, genetic predisposition, abnormal cellular metabolism and signalling, infections, environmental pollution, and an unhealthy lifestyle [5, 41, 68, 78, 94, 104]. Inherited genetic defects, for instance, mutations in certain tumor suppressor genes can increase the risk of cancer development [9]. Some of the inheritably received genetic flaws (such as mutations in BRCA1 or BRCA2) and infectious diseases may raise the risks of cancer. Environmental pollution, irradiation or poor lifestyle, for example, smoking can enhance DNA damage and thus can lead to cancer [4, 49, 51, 94, 104]. Damaged DNA can be repaired by cellular DNA repair machinery, and in case of severe DNA insult, if the repair mechanism fails, cells are led to death by apoptosis [24, 29, 47, 92]. When the damaged cells evade the DNA repair mechanisms and apoptosis, they grow in an uncontrolled manner and become cancerous [45, 52, 104, 123].

The PI3K (phosphatidylinositol 3 kinase) signaling pathway has a very important role in carcinogenesis [117]. Activating mutations in the PIK3CA (phosphatidylinositol-4,5-bisphosphonate 3-kinase, catalytic subunit alpha polypeptide) gene—through the PI3K/AKT/mTOR signaling pathway—induce the synthesis of cyclooxygenase 2, which in turn establishes the formation of prostaglandins. Cyclooxygenase 2 and prostaglandin E2 have a strong angiogenic, antiapoptotic effect favoring the growth and survival of tumor cells [30]. PIK3CA mutations are detected in approximately 40% of estrogen receptor (HR+) and epidermal growth factor receptor 2 (HER2-) breast cancers [6]. This mutation induces excessive activation of the alpha isoform of the enzyme PI3K (phosphatidyl inositol 3 kinases) [28]. This is part of an intracellular signaling pathway involved in the development of tumors and the emergence of resistance to oncological treatments. This PIK3CA mutation found in patients with breast cancer has a much lower response to tyrosine kinase inhibitors, such as lapatinib and trastuzumab. Also, the mutation has a predictive value on the response to adjuvant hormone therapy [28].

A large body of studies has reported that dysregulations in PI3K/mTOR are associated with the development of various types of cancer in humans [16, 17, 57, 83, 98, 109]. Because of the strong association in cancer, studies are being carried out to develop the inhibitors of PI3K/mTOR to treat different types of cancer. The mTOR (mechanistic/mammalian target of rapamycin) pathway was first discovered in late 1970 after the isolation of the mTOR inhibitor, rapamycin [42, 77, 107]. The mTOR inhibitors are a family of compounds that are being used for treating several human diseases such as cancer, autoimmune diseases and neurodegeneration. mTOR is a threonine/serine kinase which belongs to the family of phosphoinositide 3-kinase-related kinase (PI3K). Dysregulation of mTOR signaling has been reported to be associated not only with cancer but also with autoimmune disease, obesity, neurodegeneration, infectious diseases, and ageing [26, 83, 98, 109]. Arresting mTOR signaling with specific inhibitors, for instance, rapamycin and rapalogues are being studied extensively in both clinical and preclinical settings for better treatment of these different diseases.

Recent phase II clinical studies with rapamycin for the treatment of multiple sclerosis have revealed promising outcomes [12]. To limit the potential undesired side effects of current mTOR inhibitors, it is important to identify more potent novel targets. ATP competitive inhibitors of mTOR, for example, OSI-027 and its analogues are promising anticancer drugs [74]. Furthermore, recently revealed crystal structures of the mTOR complex would provide new insights for the advancement of more powerful and efficient mTOR inhibitors in future. The clear-cut efficacy of rapamycin and rapalogues in multiple therapeutic settings has propelled interest to discover new types of inhibitors that may be more potent and eventually with fewer side effects than rapamycin and rapalogues that include ATP competitive mTOR inhibitors. The current review summarizes the use of sirolimus and its derivatives and addresses potential limitations in targeting mTOR signaling for the treatment of cancer.

## Methodology

This comprehensive and up-to-date analysis highlights pharmacological uses as potential cytostatic agents of sirolimus and its derivatives in controlling mTOR signaling for cytostatic therapy. The data were obtained by analyzing databases in the electronic scientific literature, including online databases for medicine: Pubmed/Medline, Web of Science, TRIP Database, Scopus, Google Scholar, SciFinder, Clinicaltrials.gov, using the next MeSH terms: “Antineoplastic Agents/pharmacology”, “Antineoplastic Agents/therapeutic use”, “Drug Resistance”, “Neoplasm”, “Humans”, “Molecular Targeted Therapy/methods”, “Neoplasms/drug therapy”, “Neoplasm Proteins/antagonists & inhibitors”, “Neoplasms/metabolism”, “Protein Kinase Inhibitors/pharmacology,” “Protein Kinase Inhibitors/therapeutic use”, “Signal Transduction/ drug effects”, “TOR Serine-Threonine Kinases/antagonists & inhibitors”, “Serine-Threonine Kinases/metabolism”. The most important pharmacological data have been summarized in tables and figures.

## mTOR inhibitors: chemistry and mechanistic perspectives on cancer

**Fig. 1:**
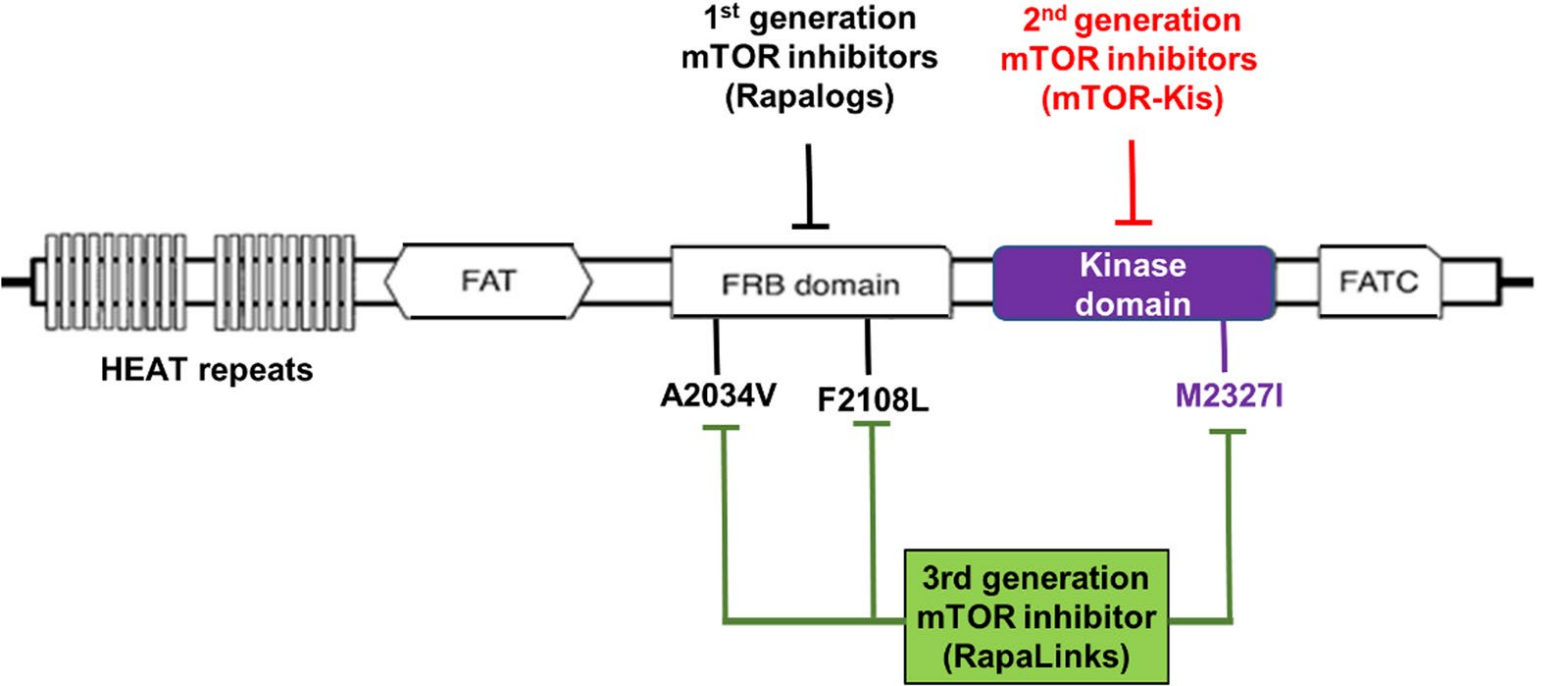
Schematic representation of different domains of mTOR and the inhibitors where those binds

The pharmacological target of FK506-binding protein 12-rapamycin-associated protein 1 (mTOR) is made up of 2549 amino acids with many structural domains (Fig. 1. HEAT (presence of anti-parallel helices in the elongation factor 3, Huntingtin, TOR1, and PP2A) repeats, FAT (for FRAP, ATM, TRAP), FATC (for C-terminal FAT) domains, kinase, and FRB are examples. As shown in Fig. 1, HEAT repeats are positioned at the N-terminal of mTOR and are necessary for mTOR multimerization. mTOR binds to FRB-FK506 binding protein 12 (FKBP12) and rapamycin via FRB-FK506 binding protein 12 (FKBP12)-rapamycin binding-domain. FAT, FATC domains and kinase are all necessary for PIKK activity in phosphatidylinositol 3-kinase-related kinases (PIKKs) [71].

To suppress mTOR activity, mTOR inhibitors of the first-generation interact with FKBP12, which further binds to the FRB domain of mTOR. Second-generation mTOR inhibitors work as ATP-competitors by competing with ATP molecules for attaching to the mTOR kinase domain. The third generation of mTOR inhibitors is a more recent family of inhibitors that are developed to be active against drug resistance in cancer cells with mTOR FRB/kinase domain mutations [75].

### The first generation mTOR inhibitors

**Fig. 2:**
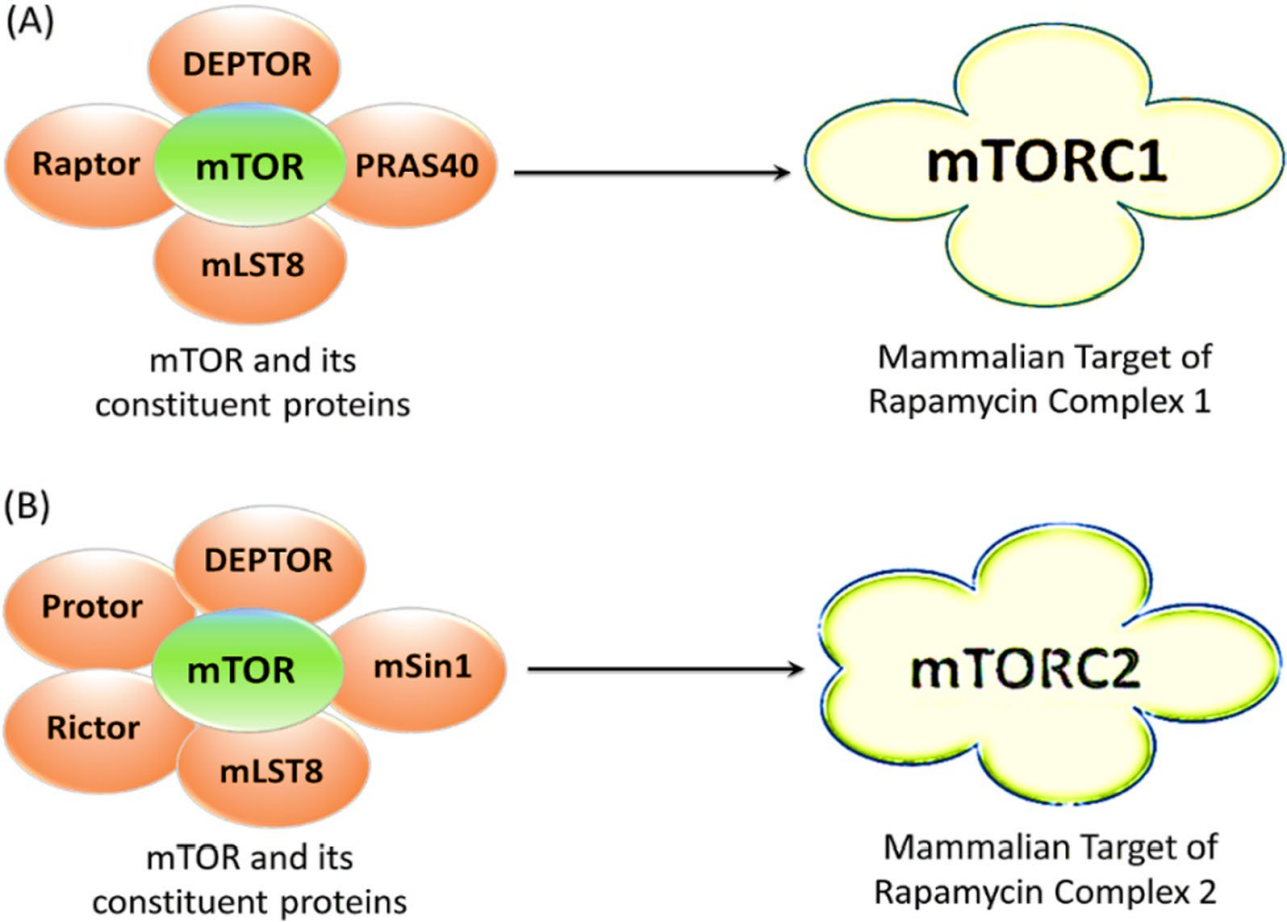
Constituent proteins of mTORC1 and mTORC2. **A** a central signaling molecule that is the mammalian target of rapamycin (mTOR) serves as a core constituent to form a distinctive complex with other molecules DEPTOR, PRAS40, mLST8 and Raptor, resulting in the formation of the mTORC1 complex, while the **B** mTOR with other five proteins DEPTOR, mSin1, Mlst8, Rictor and Protor forms the mTORC2 complex. Both the distinct protein complexes regulate several cellular mechanisms

Rapamycin, the prototype mTOR inhibitor, was originally used for over two decades as an immunosuppressant, preventing T-cell activation. Rapamycin has a selective immunosuppressive action by inhibiting the stimulation of T cells induced by some stimuli, blocking the intracellular signaling, dependent and independent of calcium. The research results have shown that the immunosuppressive mechanisms of rapamycin are other than the mechanisms of action of ciclosporin, tacrolimus and other immunosuppressive agents. Preclinical pharmacological studies suggest that rapamycin binds to the specific cytosolic protein FKPB-12, and the FKPB-12/rapamycin complex blocks activation of mTOR, a kinase critical for cell cycle progression. By blocking mTOR, specific pathways of intracellular signal transduction are inhibited. The final effect is to stop the activation of lymphocyte cells, which generates immunosuppression. In vivo, rapamycin has a direct effect on immune-mediated responses to suppress T- and B-cell activation, such as allograft rejection [67]. Rapamycin, on the other hand, does not directly block mTOR kinase activity. Instead, it binds to mTORC1, in a domain close to the active site of the kinase, but not to mTORC2 [13, 19]. As a result, it only inhibits certain of mTORC1's actions. The main components of mTORC1 and mTORC2 are shown in Fig. 2 [40, 80].

According to a recent study, the binding of rapamycin-FKBP12 and mTOR does not disrupt the mTORC1 dimer, but it does restrict access to the active site cleft from 20 to 10, showing that the FRB domain works as a barrier to the binding site of an active substrate [11]. Although rapamycin is extremely selective for mTOR, it does not effectively block all mTORC1 [101] actions and may inhibit mTORC2 in certain cell types when therapy is continued for a long time [81]. Though rapamycin does not directly interact with mTORC2, attaching with mTOR in a complex form with FKBP12, it can indirectly affect mTORC2 [114]. The prototype rapamycin's pharmacokinetic properties are not ideal. This prompted the additional study, which resulted in the discovery of rapamycin analogues (also known as rapalogs) with superior 'drug-like' effects. Several such compounds have been published in the literature, demonstrating their efficacy in the treatment of disorders such as cancer. These include RAD001 (everolimus, created by Novartis) [86, 87], CCI-779 (temsirolimus, developed by Wyeth-Ayerst/Pfizer), and AP23573 (ridaforolimus, developed by Merck/Ariad). Novartis recently demonstrated several semi-synthetic rapamycin analogues [2], that are mTORC1 inhibitors which have the potential to treat a variety of illnesses and disorders, including cancer, transplant rejection, neurological disorders, inflammation, autoimmune diseases, age-related disorders, fungal infections, and many more. These semi-synthetic rapamycin analogues have typically been derivatized at different skeletal carbons of the macrolide ring (such as C16, C32, and C40) to improve aqueous solubility, oral bioavailability, and other pharmacokinetic features [2].

Temsirolimus was authorized by the US Food and Drug Administration in 2007 for treating advanced-stage renal cell cancer. Temsirolimus is a selective mTOR inhibitor, it binds to the intracellular protein FKBP-12, and the complex FKBP-12/temsirolimus binds to mTOR, which controls the division of cancer cells, thus inhibiting its activity*. *In vitro experimental studies, showed that at high concentrations, temsirolimus binds to mTOR inhibiting its activity in the absence of FKBP-12. Also, the results of the studies showed a biphasic, dose-dependent response for cell growth inhibition. High concentrations resulted in complete inhibition of cell growth in vitro, whereas inhibition mediated only by the FKBP-12/temsirolimus complex led to a decrease of approximately 50% in cancer cell proliferation. Therefore, inhibition of mTOR activity causes (i) growth delay in the G1 stage at nanomolar concentrations; (ii) growth interruption at micromolar concentrations in the treated tumor cells, as a result of the selective interruption of the protein translation process cell cycle regulators such as D-type cyclins, c-myc and ornithine decarboxylase. When mTOR activity is inhibited, its ability to phosphorylate is blocked, and it implicitly controls the activity of the protein translation factors 4E-BP1 and S6K and the PI3 kinase/AKT metabolic pathway that controls cell division. In addition, mTOR also regulates the translation of inducible factors by hypoxia, HIF-1 and HIF-2 alpha. These transcription factors regulate the tumor's ability to adapt to hypoxic microclimates and to produce vascular endothelial growth factor (VEGF), with an angiogenic role. Therefore, the antitumor effect of temsirolimus can be attributed, in part, to its ability to decrease HIF and VEGF values in the tumor or the tumor microclimate, thus affecting tumor vascular development.

Everolimus (RAD001) has since been utilized as a single chemotherapeutic drug as well as in combination for different malignancies, including HER2-positive breast cancer and neuroendocrine tumors [54]. Everolimus is also a selective mTOR inhibitor which binds to the intracellular protein FKBP-12 and forms a complex that inhibits the activity of the mTOR-1 complex (mTORC1). Inhibition of the mTORC1 signaling pathway interacts with ribosomal protein translation and synthesis by decreasing activity of protein kinase S6 at the level of ribosomes (S6K1) and the protein-binding eukaryotic elongation factor 4E (4EBP-1) that regulate proteins involved in the cell cycle, angiogenesis and glycolysis. S6K1 phosphorylates estrogen receptor activator function domain 1, which is responsible for ligand-independent receptor activation. In vitro and in vivo studies have shown that Everolimus reduces the levels of VEGF involved in angiogenesis in cancer cells. Also, it is an important inhibitor of the growth and proliferation of tumor cells, endothelial cells, fibroblasts, vascular smooth muscle cells and reduces glycolysis in solid tumors.

Despite their powerful activity, rapamycin and rapalogs have not been used to their full therapeutic potential. The limitations of therapy with rapalogs derive from possible interactions with some CYP3A4 inhibitors that can decrease their metabolism, increasing their blood levels. For example: antifungals (clotrimazole, fluconazole, voriconazole), antibiotics (clarithromycin), protease inhibitors (ritonavir, telaprevir). Adverse effects of immunosuppressants such as infections, nervous system, cardiac or gastrointestinal disorders may also occur.

### Second-generation mTOR inhibitors

Because rapamycin has a limited ability to regulate all actions of mTORC1, and thus its application in cancer treatment, a great deal of research has gone into developing compounds that can block the catalytic activity of mTOR. These can block all phosphorylation processes mediated by mTORC1, but they will also affect mTORC2. On average, half of the maximal inhibitory concentration (IC_50_) of these inhibitors against mTOR function is significantly lower than that of PI3K. Because suppression of mTORC1 and mTORC2 may result in stronger effectiveness than mTORC1 inhibition, this class of inhibitors might be a better alternative to rapalogues for cancer therapy.

A group of researchers studied in vitro the mTOR inhibitors PP242 and PP30, which have a central pyrazolo[3,4-d]pyrimidine ring with a C4 amino group, two different heterocyclic substituents, and an N1 isopropyl substituent on C3 [7]. With IC_50_ values of 8 nM and 80 nM, correspondingly, these drugs demonstrated remarkable selectivity for mTOR among a panel of 219 kinases. Both inhibited mTORC1 and mTORC2 in an ATP-competitive manner and had greater impacts on cell cycle, cell growth and proliferation, and cap-dependent translation rather than the prototype inhibitor rapamycin [7]. Following a high-throughput screen and subsequent lead optimization campaign, Pfizer-Wyeth researchers identified WYE-354, WAY-600, and WYE-687 as effective ATP-competitive mTOR inhibitors with identical pyrazolo[3,4-d] pyrimidine moiety as core functional moiety [119]. These compounds have a 4-piperidinyl-1-substituted moiety in N1 and a C4 morpholino substituent, and in enzymatic studies, they suppress mTOR with IC_50_ values in a range of 5–9 nM, with great selectivity (greater than 100-fold) against PI3Ks. In contrast to rapalogs, in vitro, they reduced phosphorylation of mTORC2 and mTORC1 substrates in response to amino acids, growth factors as well as PI3K/Akt overexpression. Structure–activity relationships exploration of the lead molecules, particularly modification on piperidine ring and functionalization of carbamate and urea groups on C6 phenyl, resulted in the revelation of highly potent and selective mTOR inhibitors, for example, compounds 6–9 with an IC_50_ value of 0.5 nM against mTOR as determined by an enzymatic assay [119].

**Fig. 3:**
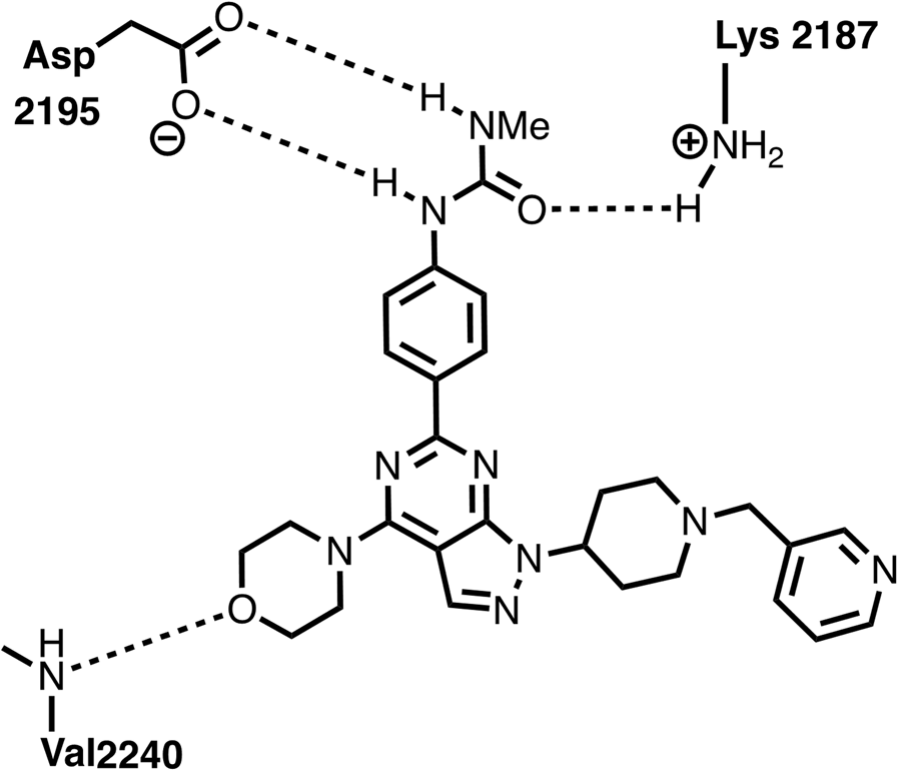
Proposed origin of potency and selectivity of pyrazolopyrimidine analogs for mTOR

Using the X-ray crystal structured pyrazolopyrimidine inhibitor interacts with PI3K and molecular docking studies with the mTOR homology model, it was discovered that 3 hydrogen bonds may be created between the pyrazolopyrimidine inhibitor and the mTOR ATP-binding pocket [106]. According to Fig. 3, there could be two H-bonds between Asp2195 and urea NHs and, one between Lys2187 and the carbonyl of the urea.

Following the identification of the pharmacophore responsible for mTOR inhibition, tremendous effort was expanded in the quest for selective mTOR inhibitors [50, 120]. The central pyrazolopyrimidines structures were extended to thienopyrimidines and triazines core structures, and multiple publications on strong mTOR inhibitors based on these structural motifs were reported.

Torin 1 was first disclosed by Nathanael Gray's group [100] and subsequently developed by AstraZeneca. This drug has a low nanomolar IC_50_ against mTOR and a 100-fold selectivity over other kinases in vitro*.*

Ku-0063794 from KuDOS Pharmaceuticals which is now a part of AstraZeneca is another example of an ATP-competitive mTOR inhibitor with strong anti-proliferative activity against cancer cells in vitro [37]. AstraZeneca researchers later used Ku-0063794 to produce AZD8055, an orally accessible version of the former having antiproliferative action and an IC_50_ of 50 nM [27, 95].

XL388 is another selective small-molecule ATP-competitive mTOR inhibitor having 8 nM and 166 nM IC_50_ respectively, that inhibits mTORC1 and mTORC2 in vitro [61]. In MCF-7 cells, this candidate effectively inhibits mTORC1 phosphorylation of p70S6K (Thr389) and mTORC2 phosphorylation of Akt (Ser473). It was found to be particularly effective in solid as well as hematopoietic cell lines of tumor in combination with paclitaxel/carboplatin and doxorubicin or as a single drug.

**Fig. 4:**
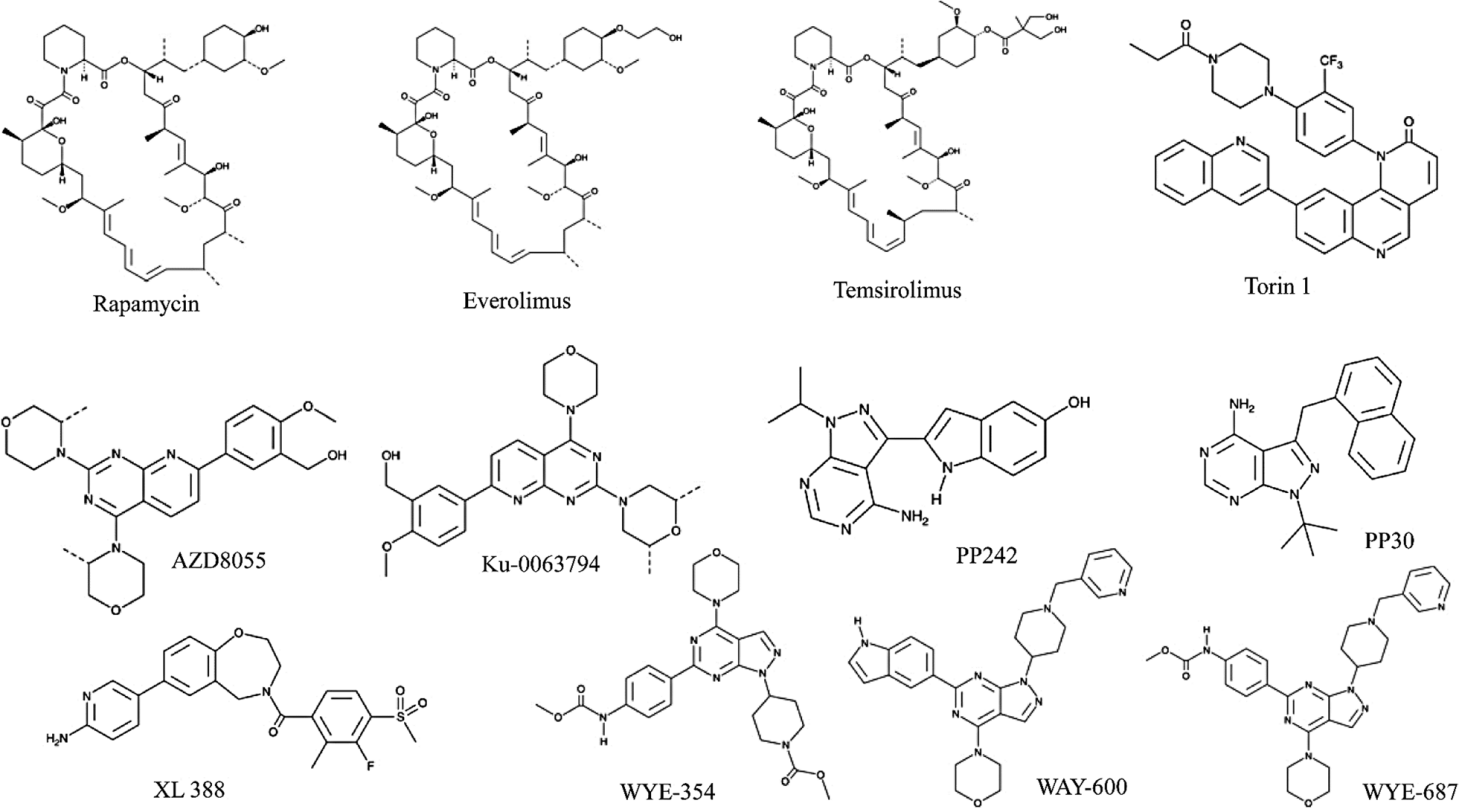
Chemical structures of mTOR inhibitors

The chemical structures of the most representative mTOR inhibitors are summarized in Fig. 4.

### mTOR inhibitors in clinical studies

**Fig. 5:**
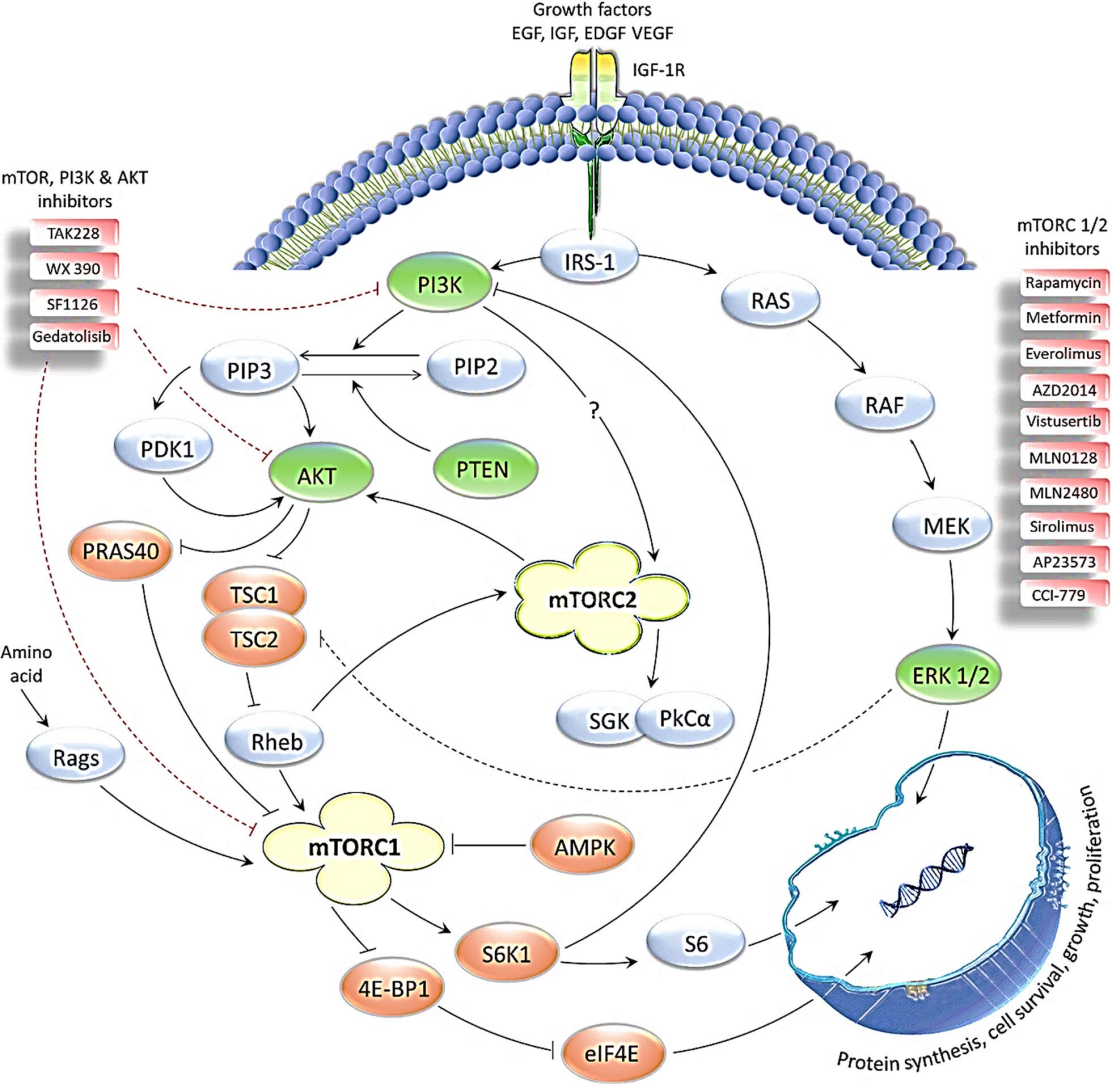
The mTORC1/2 signaling pathway and its inhibitors in clinical trials

Because of sirolimus's efficacy in preclinical studies, sirolimus-derived compounds have now been proposed for use in several clinical studies as anti-cancer medicines (see Table 1; Fig. 5 for a summary).

**Table 1:** The inhibitors for mTORC1/2 complexes that are being tested alone or in combination with other therapeutics in different phases of clinical trials for several known malignancies

mTOR inhibitor	Combinational therapy	Type of cancer/diseases	Clinical status	References/ClinicalTrials ID
AZD2014	N/A	Glioblastoma Multiforme	Phase 1	NCT02619864
AZD2014	Anastrozole	Hormone Receptor-Positive endometrial carcinoma	Phase 1 & 2	NCT02730923
AZD2014	Olaparib and AZD5363	Breast Cancer Malignant Female Reproductive System Neoplasm	Phase 1 & 2	NCT02208375
Everolimus (RAD001)	N/A	Prostate Cancer Patients with Detectable PSA Following Prostatectomy	Phase 1	NCT01548807
AZD2014	N/A	NF2 Patients with Progressive or Symptomatic Meningiomas	Phase 2	NCT02831257
Vistusertib (AZD2014)	N/A	Recurrent Grade II-III Meningiomas	Phase 2	NCT03071874
Everolimus	Levonorgestrel-Releasing Intrauterine System	Atypical Hyperplasia or Stage IA Grade 1 Endometrial Cancer	Phase 2	NCT02397083
AZD2014	Rituximab	Relapsed/Refractory Diffuse Large B Cell Lymphoma	Phase 2	NCT02752204
MLN0128	MLN1117 oral inhibitor of the PI3K (alpha) isoform	Advanced Nonhematologic Malignancies	Phase 1	NCT01899053
Milled MLN0128 API	Unmilled MLN0128 API and Paclitaxel	Advanced Nonhematologic Malignancies	Phase 1	NCT02412722
MLN2480	MLN0128 or Alisertib, or Paclitaxel, or Cetuximab, or Irinotecan	Advanced Nonhematologic Malignancies	Phase 1B	NCT02327169
AZD2014	Paclitaxel	Ovarian cancer Squamous cell lung cancer	Phase 1	NCT02193633
TAK228	Paclitaxel	advanced/Recurrent Epithelial Ovarian, Fallopian Tube Primary Peritoneal Cancer	Phase 2	NCT03648489
Sirolimus	N/A	Cardiovascular Abnormalities/Vascular Malformations	Phase 3	NCT01811667
AP23573 (Ridaforolimu)	N/A	Advanced Sarcoma	Phase 2	NCT00093080
Rapamycin	Placebo	Aging and associated complications	Phase 2	NCT02874924
Everolimus	Imatinib mesylate	Metastatic or Unresectable Kidney Cancer	Phase 2	NCT00331409
MLN0128	Paclitaxel; Trastuzumab	Advanced Solid Malignancies Hematologic Malignancies	Phase 1	NCT01351350
WXFL10030390	N/A	Advanced Solid Tumors Lymphoma	Phase 1	NCT03730142
Metformin	N/A	Well-differentiated Neuroendocrine Tumors	Phase 2	NCT02279758
SF1126	N/A	Advanced or Metastatic Solid Tumors	Phase 1	NCT00907205
Everolimus	N/A	Chronic Allograft Dysfunction in Renal Transplantation	Phase 4	NCT01046045
Sirolimus	N/A	Congenital Vascular Malformations	Phase 3	NCT03987152
Sirolimus	N/A	Peutz-Jeghers Syndrome	Phase 4	NCT03781050
RAD001 (Everolimus)	N/A	Tuberous Sclerosis Lymphangioleiomyomatosis	Phase 1 & 2	NCT00457964
RAD001 (Everolimus)	N/A	Subependymal Giant Cell Astrocytoma Tuberous Sclerosis	Phase 1 & 2	NCT00411619
RAD001 (Everolimus)	N/A	Epilepsy Tuberous Sclerosis Complex	Phase 1 & 2	NCT01070316
Sirolimus	Placebo	Polycystic Kidney, Type 1 & Type 2 Autosomal Dominant Disease	Phase 3	NCT02055079
Sirolimus	N/A	Blue Rubber Bleb Nevus Syndrome Hereditary Sporadic Venous Malformation	Phase 4	NCT03767660
Gedatolisib	Palbociclib/Letrozole Or Palbociclib/Fulvestrant	Metastatic Breast Cancer	Phase 1B	NCT02684032
Arm 1 Everolimus/ tacrolimus	Calcineurin inhibitors	Renal Transplant and associated complications	Phase 4	NCT01935128; [80]
RAD001 (Everolimus)	Placebo	Tuberous Sclerosis Complex (TSC) Lymphangioleiomyomatosis (LAM)	Phase 3	NCT00790400; [80]
CCI-779 (Temsirolimus)	N/A	Breast and Renal cancer	Phase 2	[73]

ClinicalTrials ID has been taken from https://clinicaltrials.gov

Temsirolimus (CCI-779; Wyeth-Ayerst), deforolimus (AP23573; Ariad Pharmaceuticals), and everolimus (RAD001; Novartis) are three analogues currently being studied [3, 34, 69]. All three drugs, like sirolimus, work by creating complexes with FKBP12, that link to and further suppress mTOR. They've been restructured to improve water solubility and stability. The C40 hydroxyl of sirolimus is typically substituted with esters or ethers in synthetic modifications [32]. Temsirolimus is an ester derivative of sirolimus that can be given intravenously or orally, whereas everolimus is a hydroxyethyl ether derivative that can be given orally. Deforolimus is a phosphonate replacement that can be given intravenously or orally [32, 103]. When taken according to the right cancer treatment plan, sirolimus analogues do not cause immunosuppression.

Temsirolimus was originally tested in patients with solid tumors for example breast, lung, and kidney malignancies in phase I clinical studies. Temsirolimus was delivered intravenously once daily for 5 days every 2 weeks or once weekly, according to two different dosing regimes [46, 73]. During these two investigations, 87 patients were given temsirolimus. Three partial responses (PRs are well-defined as at least a 50% decrease in overall tumor size) were found, one for kidney, one for breast, and one for lung tumors. Two patients with renal cancer achieved minor responses (reductions of tumor of 34% and 39%, respectively), and two patients had disease stability for longer than 6 months. As a result of these findings, a couple of clinical phase II studies for temsirolimus was started.

In one phase II trial of 109 patients with primary advanced or metastatic breast cancer, 10 had PRs (a 9.2% response rate) [25]. Temsirolimus treatment resulted in one complete response and seven PRs in 111 individuals with renal cell cancer (7% response rate). [10]. Temsirolimus has also shown promising results in treating endometrial cancer. In another phase II study of individuals with recurrent or metastatic endometrial carcinoma, 26 percent (5 of 19 patients) had PRs, while 60 percent had stable disease (12 of 19 patients). Furthermore, in phase II trials, temsirolimus showed a remarkable potential for mantle cell lymphoma [112]. In addition, temsirolimus is the first sirolimus-derived compound to go through phase III clinical trials for effective renal cancer therapy. Patients who received temsirolimus as a single intravenous agent had a significantly higher median survival of 10.9 months than patients who received the standard cancer therapy of interferon- [IFN] (7.3 months) [79]. In May 2007, the US FDA authorized temsirolimus for treating advanced renal cell carcinoma based on its efficacy in this phase III trial [3, 31].

In phase, I investigations of everolimus for treating solid tumors, oral dosages of 20–30 mg on weekly basis were devised, and further suppression of S6K in peripheral mononuclear cells of blood was found as an alternate measure for therapeutic action [22]. Phase II clinical studies of Everolimus in individuals with renal and endometrial cancer has completed recently and a hematological malignancy phase I/II study has been done [8, 97]). Everolimus was given orally to 27 individuals with a range of hematological malignancies (such as mantle cell lymphoma, acute myelogenous leukemia, and B-chronic lymphocytic leukemia). Two patients had favorable hematological responses, while mTOR signaling inhibition was tested in 9 patients, with 6 showing a reduction in S6K and/or 4E-BP1 phosphorylation [118]).

A phase III clinical study of Everolimus in people with advanced metastatic carcinoma of the renal cell was recently investigated [64]. Throughout the research, 272 participants were given a single oral dose of everolimus daily (10 mg). In general, everolimus therapy was usually well tolerated, with 63 percent of patients (171 out of 272) demonstrating disease stability (a disorder that stayed constant for a minimum of 56 days), demonstrating that everolimus is an efficient treatment choice for advanced carcinoma of the renal cell.

Clinical testing for Deforolimus is now in its early phases, while several phase I and phase II clinical trials have already been over. Deforolimus was administered intravenously daily in a phase I trial for 5 days in every 2 weeks in individuals with resistant or advanced solid tumors [62]. mTOR suppression, as evidenced within 4 h after deforolimus treatment, as revealed by dephosphorylation of 4E-BP1 in 12.5 percent of patients (four of 32 patients) [62]. Furthermore, in phase II trials of deforolimus, it has been studied in patients with advanced-stage sarcomas as well as resistant hematological malignancies (through intravenous administration); nevertheless, preliminary data show poor objective response rates [97]. Deforolimus decreased mTOR signaling in patients with high-grade sarcomas, as evidenced by a reduction in the amounts of the ribosomal protein S6 which is being phosphorylated in tumor sections [31, 48].

The upstream signaling molecules that play a crucial role in mTORC1/2 signaling are the PI3K, PTEN and AKT. Both of the mTORC1/2 complexes are important in cellular growth, survival, proliferation, motility, protein synthesis and autophagy. All of the inhibitors depicted here are currently being tested in different phases of clinical trials for several disorders, majorly for different types of cancers. TAK228, an oral inhibitor has been developed to inhibit PI3K/AKT/mTOR, while WX390, SF1126 and Gedatolisib are reported to target PI3K and mTOR. All of the remaining inhibitors are being evaluated in clinical trials for mTORC1/2 inhibition. The figure is adapted from [40, 76, 80].

## Therapeutic perspectives, limitations and challenges associated with targeting mTOR

Cancer treatment has evolved rapidly over the last decade, toward a personalized approach [47, 89]. Modern technologies today allow a molecular characterization that outlines a unique picture for each patient. Based on tumor genomic changes, new treatment targets have been discovered, some of which can be acted upon directly through personalized therapies. Tumor genomic testing has evolved, from several biomarkers to extensive panels—allowing the analysis of all mutations that can be acted upon by targeted therapies, and even more, biomarkers that allow patients to be included in clinical trials for treatments not yet approved. Regarding the therapeutic targets in cancer, research in this field has led to the approval of many drugs in recent years and there are many other molecules still in clinical or preclinical studies, so we can expect complex changes in therapeutic standards in cancer in the near future [72].

Although their initial success, ATP-competitive mTOR inhibitors have yet to reach their therapeutic potential for a variety of reasons such as:

i. Inhibiting mTOR activates a variety of feedback loops targeting upstream signaling pathways, which boost cancerous cell survival and further metastasis when activated [102].
ii. Because mTOR signaling is essential for normal cell function, the total blockage is extremely harmful to healthy tissues [124].
iii. mTORC1 inhibits autophagy, and treatment with an mTOR inhibitor may induce autophagy, thus promoting cancer cell survival, as seen with AZD8055[99].
iv. Any clinically relevant mTOR mutations that increase mTOR's catalytic activity could drastically diminish the efficiency of such inhibitors in cancer cells [44].

**Fig. 6:**
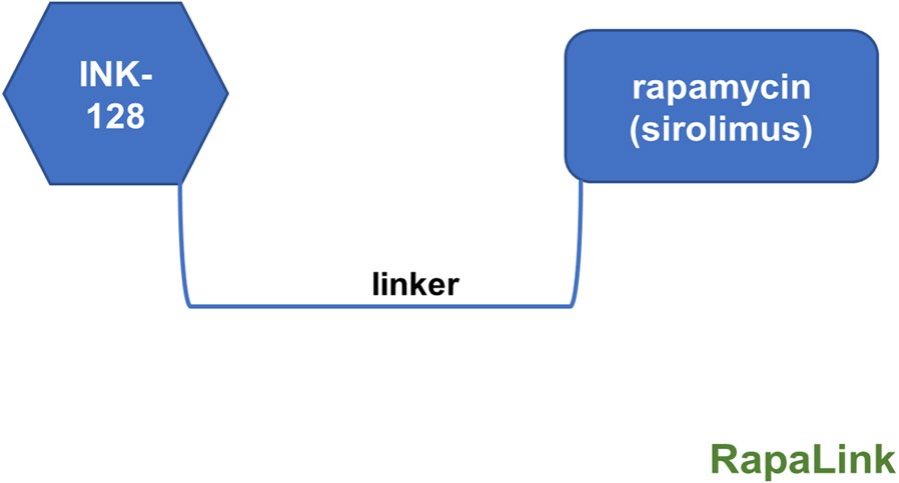
Generation of RapaLinks. Linking an mTOR kinase inhibitor INK-128 (or MLN0128) to rapamycin led to RapaLinks which exhibited improved efficacy in tumor-bearing mice than each of the constituents alone

To solve this issue, Rodrik-Outmezguine has effectively linked the rapamycin and INK-128 binding sites leading to the generation of a bifunctional mTOR inhibitor called RapaLink [75]. This hybrid molecule now contains both rapamycin and an mTOR kinase inhibitor, which are linked via a non-perturbing, strain-free crosslinker of optimal length. The linker permits the chemical to connect with the FRB domain of mTOR by interacting with FKBP12, along with reaching the kinase domain of mTOR, allowing it to serve as an ATP-competitive inhibitor (Fig. 6). Both RapaLinks (1 & 2) inhibited mTORC1 and mTORC 2 with IC_50_ of 10 nM, and mice xenografts of MCF-7 cells were shown to be more sensitive to RapaLink-1 than parent rapamycin and INK-128. Furthermore, after 9 months of treatment, RapaLink-treated cells did not acquire chemotherapeutic drug resistance, but considerable resistance was identified after 3 months of treatment with first- or second-generation mTOR inhibitors. This discovery opened the way for developing a new generation of mTOR inhibitors.

The clinical use of mTOR inhibitors has also shown other effects. For example, some studies conducted on mice showed that Sirolimus extended their life almost three times [21]. Rapalogs were approved by the FDA in the early 2000s and some research has suggested them as potential antiaging drugs [88]. If more than ten years ago Sirolimus was used as an immunomodulator, in high doses it was observed that it can act as an immunostimulator, especially in elderly patients with oncological diseases [33]. In addition, some studies have indicated that in patients with cardiovascular diseases to whom stents were applied, the administration of Rapamycin reduces the restenosis rate of the stents [59]. Other research has shown that mTOR inhibitors can induce some metabolic and stress responses that promote longevity, although exactly how this happens is still not clear [21].

Despite the efficacy of sirolimus derivatives in preclinical research as anti-cancer drugs, it is crucial to remember that inhibitors of mTOR have not shown to be as efficient as predicted. Based on this, sirolimus as a wide-ranging monotherapy for treating cancer may be unsuccessful. As a result, determining which patients might benefit the most from sirolimus medication is crucial and exploring sirolimus as part of combination medicines for cancer treatment [31]. In addition to the compounds mentioned above, recently evolving compounds that regulate/inhibit mTORC signalling, and its associated components, and might be useful for the treatment of various types of cancers are summarized in Table 2.

**Table 2:** Evolving compounds and reagents regulating or inhibiting mTOR signaling, and its associated components, in various cancers

Tested compounds	Preclinical study/mechanisms	Refs.
RMC-4627	In vitro models of B-cell acute lymphoblastic leukemia RMC-4627 BCR-ABL ↓4E-BP1 phosphorylation ↓ cancer cells progression ↓viability ↓ cancer cell survival	[55]
1,4-O-diferuloylsecoisolariciresinol (IM-1)	In vitro mice embryonic fibroblast cells ↑nuclear translocation ↑S6K kinase ↑4E-BP1 ↑cytotoxicity ↑apoptosis	[116]
Pierreione B (IM-2)		
DL001	In vitro PC3 cells MEFs mice embryonic fibroblasts ↓hyperactive mTORC1 In vivo C57BL/6J mice ↓side effects of rapalogs	[85]
DHM25	In vitro triple-negative breast cancer cells ↓Akt phosphorylation	[35]
3HOI-BA-01	In vitro non-small cell lung cancer cells ↓mTOR kinase In vivo mice ↓tumor growth	[113]
PF-5212384 PD-901	In vitro 14 HNSCC cell lines ↑ cells in G0-/G1 phase ↓PI3K/mTOR ↓NF-κB, ↓AP-1, ↓IL8 ↓cells proliferation, ↓apoptosis, ↓angiogenesis	[63]
P529	In vitro GBM cells ↓AKT (Ser-473),↓NDRG1 (Thr-346) ↓PKCα (Ser-657) ↓cancer cells growth, ↓invasiveness In vivo Mice GBM xenograft ↓Tumor groth	[18]
JR-AB2-011 (Palomid 529)	In vitro LLC-PK1, LLC-Mdr1a, LLC-MDR1 ↓ cancer cells’ growth In vivo WT and KO mice with gliomas ↑ blood brain passage	[56]
W922	In vitro HCT116, MCF-7, A549 ↓cancer cells viability In vivo mice xenograft model ↑ cell cycle arrest in G0-G1 phase ↑ apoptosis	[111]

The existence of the sirolimus-resistant mTOR signaling complex, mTORC2, must be considered when evaluating the usage of sirolimus derivatives for treatment. Sirolimus is considered to impair the interaction between mTOR and raptor by targeting the mTORC1 complex; however, sirolimus therapy does not affect the mTORC2 complex. As a result, in the presence of sirolimus, mTORC2 is free to signal. mTORC2 regulates the cytoskeleton, but more significantly, it is the kinase that phosphorylates Akt [15, 82]. Akt activation requires phosphorylation of Ser473 within the hydrophobic motif, as well as Thr308 phosphorylation in the activation loop [39]. Although PDK1 has long been known to phosphorylate Akt at Thr308, this has been only just exposed that mTORC2 is an enzyme that phosphorylates Ser473 in Akt [15, 82]. Rictor phosphorylation was reduced, and mTORC2 enabled Akt phosphorylation in vitro at Ser473, proving mTORC2 as the secondary kinase involved in Akt regulation, known as PDK2 [15, 82]. Because sirolimus only inhibits mTORC1 and the discovery of mTORC2 as PDK2 highlights some remarkable questions about the usage of sirolimus derivatives in cancer therapy. Because Akt is involved in numerous pro-survival and growth-promoting pathways, the continuous stimulation of Akt by mTORC2 with the combination of sirolimus in the setting of cancer is quite significant. However, some recent evidence suggests that extended sirolimus treatment inhibits mTORC2 [81]. It was postulated that after continuous sirolimus treatment, the cell compensates for mTORC1 inactivation by creating additional mTORC1 complexes, reducing the accessibility of mTOR to support the development of mTORC2. As a result, long-term sirolimus therapy blocks any beneficial signaling action relayed to Akt by mTORC2, strengthening the anti-cancer effects of mTOR inhibitors.

The signaling pathway of PI3K/Akt/mTOR is a significant regulatory mechanism that regulates a wide range of cellular processes. As a result, targeting this pathway for cancer treatment impacts key cellular processes in unpredictable ways, which might lead to mTOR inhibition resistance or perhaps a worsening of tumor development. The suppression of a negative feedback loop controlled by S6K by sirolimus is a good example. mTOR activates S6K in the presence of nutrients and growth factors. By blocking the insulin receptor substrate-1 (IRS-1) protein, S6K creates a negative feedback loop [60]. IRS-1 phosphorylation by S6K identifies it for breakdown or inhibition, resulting in decreased PI3K and Akt signaling [38, 43, 90]. However, S6K is no longer stimulated by mTOR in the presence of sirolimus or its derivatives, resulting in increased IRS-1-mediated signaling, reduced IRS-1 degradation, and elevated PI3K and Akt activity [66].

An analogous control mechanism has been reported for the platelet-derived growth factor receptor (PDGFR), in which S6K generated signal regulates PDGFR expression [121]. Disrupting these negative feedback systems has significant effects for the efficacy of sirolimus analogs in cancer therapy. For example, sirolimus treatment of cancer cells increased Akt phosphorylation (Ser473) and activation [23, 66]. Inhibition of mTOR and S6K may increase PI3K/Akt signaling, which may enhance carcinogenesis and alter tumor susceptibility to some other chemotherapeutic treatments. In a current study, O'Reilly et al. found that sirolimus therapy increased Akt phosphorylation (Ser473) and activation in cancer cells [66]. Akt phosphorylation was also shown to be higher in tumors from patients receiving everolimus medication. Skeen et al., on the other hand, revealed that despite disrupting the S6K-IRS-1 negative feedback loop, sirolimus therapy still prevented carcinogenesis [96].

Recent findings back up the usage of sirolimus in conjunction with other anti-cancer medicines. Trials utilizing the inhibitors of IGF-I/insulin signaling are already in progress [122]. In the future, it is critical to establish indicators of sirolimus sensitivity or resistance so that individuals can receive the right treatment and avoid developing chemoresistance. For establishing the utmost successful combination therapy for patients who do not have any effectiveness of conventional therapeutic procedures, more study into the probable synergism between sirolimus and standard-of-care drugs is needed [53]. A variety of difficulties concerning mTOR signaling and sirolimus action must be addressed in the preclinical context. The mTORC2 complex, for example, is poorly understood. Understanding the relevance of this signaling complex and clarifying its possible role in cancer will also need the identification of downstream targets of mTORC2 [36].

The quest for new mTOR inhibitors that aren't based on sirolimus will be a priority. Affecting the kinase domain of mTOR with novel small molecules to suppress both mTORC1 and mTORC2 activity might improve the efficacy of mTOR suppression in cancer therapy [70]. Compound 401, for example, is a novel drug that inhibits both the TORC1 and TORC2 actions of mTOR [14]. This chemical, however, is not selective for mTOR and further focuses on DNA-dependent protein kinase. It is also critical to make use of available medications that can block mTOR, like as AICAR and metformin, to get a better understanding of mTOR signaling in both normal and altered cells. More research is needed to investigate the importance and consequences of mTORC2 inhibition in tumor development. The toxicity of these compounds could be one of the most important consequences of mTOR inhibitors. For example, data from recent studies showed that the compound NVP-BEZ235 (dactolisib) has anticancer efficacy on cell lines in vitro, but on in vivo models with orthotopic glioblastoma xenograft mice, adverse effects such as alopecia, hyperglycemia, liver cytolysis [65]. Therefore, safety and toxicity studies of these compounds should be carried out in the future. The development of mTORC2 inhibitors and the potential synergism of cancer therapeutic with sirolimus derivatives is a field of research that needs to be also investigated further [31].

Also, as future perspectives, mTOR inhibitors should be considered immunosuppressive drugs that reduce or suppress the activity of the immune system [58]. They can be prescribed for the treatment of autoimmune diseases (systemic lupus erythematosus, psoriasis, rheumatoid arthritis, inflammatory bowel disease) or the prevention of graft rejection in organ transplants (liver, kidney, heart) [115]. The main advantage of immunosuppressive therapy is the improvement of the patient's quality of life [110]. Adverse reactions such as nephrotoxicity, and increased risk of malignancy or infections require careful monitoring of treatment [108]. By binding to FKBP12, sirolimus forms a complex that binds to the enzyme mTOR, which it inhibits [105]. Thus, the progression of the cell cycle from the G1 phase to the S phase is blocked. Sirolimus is combined with tacrolimus or glucocorticoids and is used to prevent organ transplant rejection. Hyperlipidemia, thrombocytopenia, anemia, oral ulcers, diarrhea, and infertility are side effects that may occur during treatments [84].

## Concluding remarks

mTOR, a serine/threonine-protein kinase, is a major regulator of several fundamental cellular functions, including development, multiplication, mRNA translation, and cytoskeletal architecture. mTOR signaling dysfunction promotes cellular development and proliferation and has been linked to a variety of human malignancies. Increased mTOR signaling is particularly related to human malignancies defined by the loss or mutation of critical tumor suppressors including STK11, TSC1/2, and PTEN which seem to be important for regulating the PI3K/Akt pathway [20]. As a result, mTOR has become a crucial cancer therapeutic target. Sirolimus and its variants are powerful and selective mTOR inhibitors that have gotten a lot of interest as possible anti-cancer drugs. For treating cancer patients, sirolimus analogues are now being studied in phase II and phase III clinical trials. So far, clinical studies show that sirolimus's performance as a single agent as a broad-range anti-cancer therapy may be rather restricted; nonetheless, certain cancers, such as endometrial carcinoma, renal cell carcinoma, and mantle cell lymphoma, respond well to sirolimus. Notably, Temisrolimus has already been approved by the FDA for treating advanced renal cell cancer.

Since the development of sirolimus occurred more than 30 years ago, much has been learnt about the significance of mTOR in cellular process coordination and its relevance in cancer. Despite recent improvements in the research of mTOR signaling in cells, notably its development as a therapeutic target for cancer treatment, more work must be done to completely comprehend the relevance of mTOR and its functions in cell biology and illness. Consequently, upcoming research will specify the insights to comprehending mTOR and its significance in medical health.

## Data Availability

Not Applicable.
